# Comparison of different types of ultrasound probes for lung ultrasound in neonates—A prospective randomized comparison study

**DOI:** 10.1371/journal.pone.0306472

**Published:** 2024-07-03

**Authors:** Lukas Aichhorn, Lisa Habrina, Tobias Werther, Angelika Berger, Erik Küng

**Affiliations:** Department of Pediatrics and Adolescent Medicine, Division of Neonatology, Comprehensive Center for Pediatrics, Pediatric Intensive Care & Neuropediatrics, Medical University of Vienna, Vienna, Austria; Shri Madan Lal Khurana Chest Clinic, INDIA

## Abstract

**Objective:**

To determine the effect of different types of probes for lung ultrasound in neonates.

**Design:**

Prospective, blinded, randomized, comparative study between 2020 and 2022.

**Setting:**

Single-center study at a third level neonatal unit.

**Patients:**

Hemodynamically stable infants with either nasal continuous positive airway pressure, high flow nasal cannula or without respiratory support.

**Intervention:**

Lung ultrasound using either an echo or microconvex probe. As control, the linear probe was used.

**Main outcome measures:**

Primary outcome measure was neonatologist performed lung ultrasound (NPLUS) score. Secondary outcome measures were number of B-Lines, thickness of the pleural line and subjective image quality. Furthermore, correlation between NPLUS results and clinical data was assessed.

**Results:**

A total of 1584 video loops from 66 patients, with a mean corrected gestational age of 33.8 weeks (SD 4.23) and weight of 1950g (SD 910), respectively, were analyzed. NPLUS score was estimated lower with the echo- and microconvex probe compared to the linear probe, with a coefficient of -2.95 (p < 0.001) and -1.09 (p = 0.19), respectively. Correlation between the pulse oximetric saturation/fraction of inspired oxygen ratio and NPLUS score was moderately strong and best using the microconvex probe (Spearman’s rho = -0.63, p<0.001).

**Conclusion:**

Our results not only confirm the current recommendations, but also demonstrate the extent of the varying results when different probes are used. The differences we discovered call for caution in interpreting scores, especially in the context of guiding therapies and communicating prognoses. Finally, the correlation between NPLUS score and clinical parameters contributes to validating the use of this diagnostic tool.

## Introduction

Lung ultrasound (LUS) has become an increasingly popular tool in recent years, particularly in neonatal and pediatric intensive care units [1,2]. Pioneered by Daniel Lichtenstein in the 1990s, LUS initially faced resistance but has since been recognized as a reliable and quick tool for detecting various lung-related pathologies. Experts in anesthesiology, radiology, neonatology, and other related fields have formulated several evidence-based recommendations for the use of LUS [3–9]. The inclusion of LUS in a POCUS guideline issued by the European Society of Pediatric and Neonatal Intensive Care (ESPNIC) can be considered a milestone in this field [10].

In our units, we try to harmonize the terminology by using the term neonatologist-performed-LUS (NPLUS) for bedside exams when performed by the attending neonatologist [11].

Over the last decade several protocols regarding NPLUS have been applied in the routine setting as it helps in the diagnosis of respiratory disorders and guides procedures such as surfactant administration and—more controversial—chest tube placement [1,12,13].

While other ultrasound applications, such as echocardiography or cranial ultrasound, have been optimized by the development of modern software and probes to increase image quality, the effect of the ultrasound probe on lung ultrasound scores and image quality remains unclear. Although a "holistic ultrasound" approach, as recommended by some authors, may be cost-effective and facilitates the clinician’s workflow, an assessment of the possible effects of different probes is still necessary [14].

Neonates and infants have less muscle, fat, and subcutaneous tissue, and comparatively cartilaginous ribs, as well as smaller, still developing lungs compared to adult patients [15]. Hence, in this patient group, the use of a linear transducer with a frequency of at least 10 MHz is recommended, and the use of a convex probe can be considered, if no linear transducer is available [16]. Therefore, it seems physiologically logical that linear transducers yield better results. However, there are few prospective studies examining the actual potential differences in the application of various probes.

Considering the various scores and their clinical associations that have been described thus far, even minor differences in a score can have significant implications. Published cut-off values exist not only for surfactant replacement therapies, respiratory assistance or extubation readiness, but also for the development of bronchopulmonary dysplasia [13,17–19]. Other studies focused on the progression of the score over time and correlate findings with clinical outcomes, including extravascular lung water [20–22].

In this study, we aim to compare the performance of three different types of ultrasound probes, namely linear, microconvex and echo (i.e., phased array) probes in hemodynamically stable neonates. Our goal is to provide insight into which probe might be the most appropriate choice for lung ultrasound in neonates, based on the current literature and our data.

## Methods

### Study design

The study was conducted as a single-center, prospective, blinded, randomized study. Examinations took place at the Department of Pediatrics and Adolescent Medicine of the Medical University of Vienna and were performed by staff physicians experienced in lung ultrasound from 12^th^ January 2020 to 22^nd^ July 2022.

The primary outcome measure is the NPLUS score, as described initially by Brat et al, while the secondary outcome measures are number of B-Lines, thickness of the pleural line and subjective image quality [23]. We furthermore correlate NPLUS results with clinical data.

This study was reviewed and approved by the local ethics committee (EK-Nr. 2167/2019). Prior registration in a clinical trials register was waived due to the diagnostic character of the intervention, no subsequent change of the routine treatment, and because no cause-and-effect relationship between an intervention and outcome was studied.

### Patients

We included hemodynamically stable infants >1000g with either nasal continuous positive airway pressure (nCPAP), high flow nasal cannula (HFNC) or without any respiratory support.

Exclusion criteria were congenital heart defects or other major malformations, presence of IVH ≥ III, and any condition that, in the opinion of the investigator, would place the neonate at risk. Written consent of a legal guardian has been obtained before performing NPLUS.

Given an anticipated 30% variation in B-Lines and NPLUS score between the different probes, a sample size of 64 patients was considered sufficient to achieve a statistical power of 0.8 and a level of significance of 0.05 using a linear multiple regression model.

### Study intervention

One of three clinicians trained in lung ultrasound conducted a standardized lung ultrasound exam using a linear probe (frequency 5 to 18 Mhz), and a supplementary examination using either a microconvex probe (frequency 5 to 8 Mhz) or echo probe (frequency 4 to 12 Mhz) which was randomly assigned prior to the exam. Simple randomization without stratification was performed using sealed envelopes.

To minimize evaluative bias, a randomly selected expert evaluator was assigned to each patient blinded to the patient’s identity, clinical state, or examination probe. The evaluation process was conducted using pseudonymized video-loops with a length of three seconds, starting with the randomized probe, followed by the linear probe, to furthermore minimize bias. Video loops were securely stored in digital format. Clinical data was obtained from the routinely maintained electronic patient file. The examination was performed by an experienced clinician with at least three years of expertise in neonatal lung ultrasound. A second person, ideally a parent, supported the infant during the exam with facilitated tucking and non-nutritive sucking in order to increase comfort.

### NPLUS protocol and scoring

NPLUS was performed with the Philips Affinity 70. Machine settings were optimized and strictly defined to ensure comparability between the different probes according to our standard and international recommendations [3,16,24]. Focus was set to the pleural line, depth to 4–5 cm, no additional filters were used, and spatial compound imaging was turned off.

In accordance with our protocol, the examination consisted of anterior, lateral, and posterior views in both superior and inferior positions, resulting in 12 views per patient.

Only longitudinal scans were evaluated [24]. The NPLUS score was assessed using the adapted method published by Brat et al. in 2015: 0 in the presence of only A-lines, 1 for ≥ 3 B-lines, 2 for coalescent B-lines, and 3 for extended consolidations for each view. Pleural thickness was measured in the right posterior inferior lung field. Even for the smaller patients, it was possible to differentiate between an upper and lower field both laterally and posteriorly with a slight position adjustment and rotation, as tolerated by the patient.

### Data acquisition and analysis

The following parameters were reported: NPLUS score, total number of B-Lines, number of B-Lines per intercostal space, consolidations, pleural line thickness, subjective visibility, and diagnostic findings. The subjective visibility score was based on a scale of 0–10 with 10 being the best visibility for the evaluator. This subjective parameter of visibility alongside the objective parameters was introduced to ensure a more comprehensive analysis of this investigator-dependent method, and a Likert-Scale from 0 to 10 was chosen to provide a detailed assessment.

Although NPLUS scoring quantitatively assesses the image, but we sought more precision and an additional measure beyond pattern recognition. Thus, the exact number of B-lines offers a more detailed analysis than the score alone. One of three different evaluators was randomly assigned to each patient to perform the analysis of the video loops.

A linear, mixed-effects model was utilized to analyze the data, considering random effects with a random intercept. The model included fixed effects of the probe, evaluator, surfactant, antenatal steroids, gestational age at birth, current age, birthweight, current weight, respiratory support, sex, inhaled budesonide treatment, state of the patent ductus arteriosus, elevated C-reactive protein and Interleukin 6 levels, APGAR Score, SF ratio, as well as blood pressure.

A p-value of less than 0.05 was considered statistically significant. Statistical analyses were performed using RStudio (version 2022.07.2). The “lmerTest”, “ggeffects”, and “ggplot2” packages were used [25–27].

## Results

**Fig 1:**
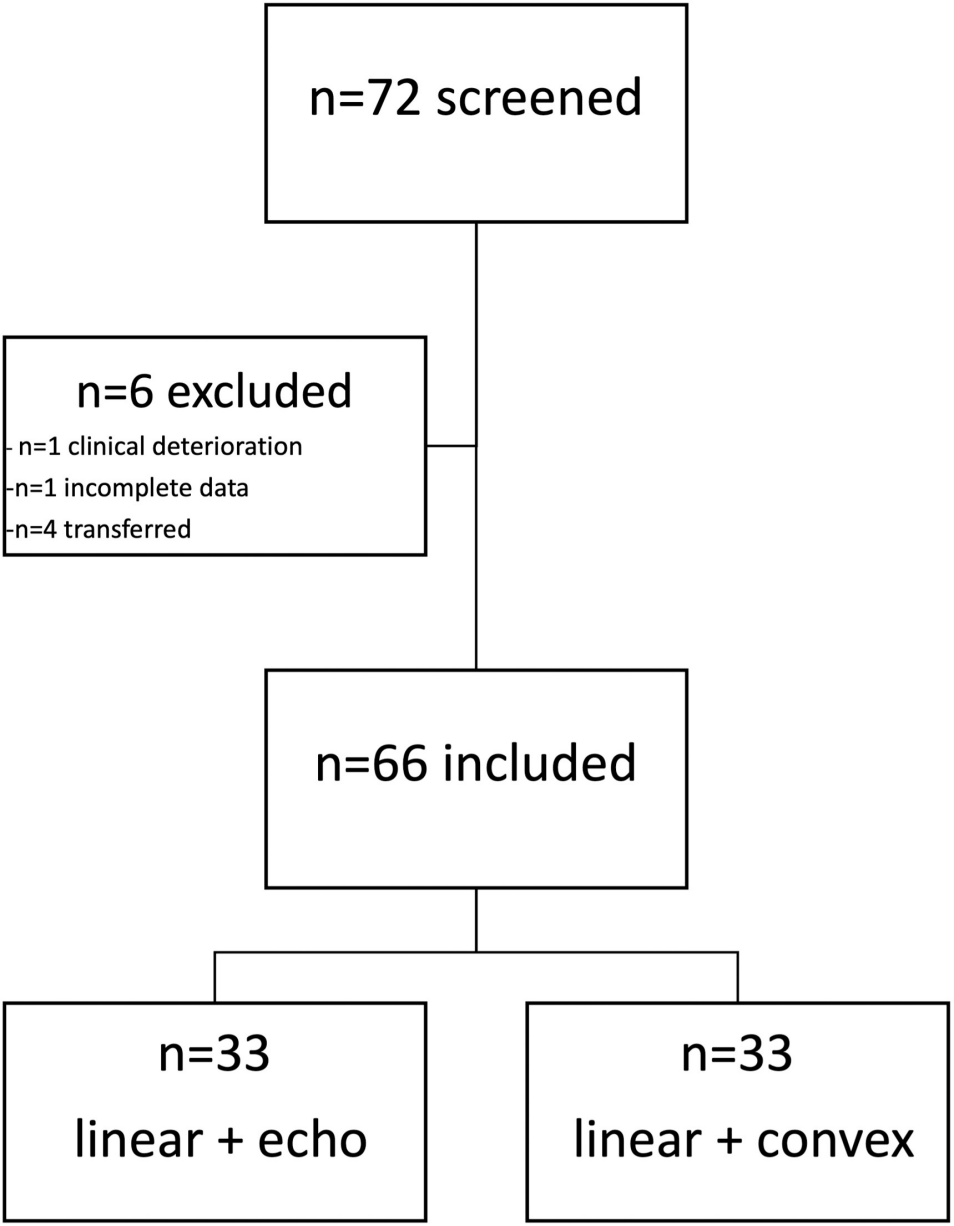
Flow chart of patient selection.

A total of 66 patients were analyzed in this study, accounting for a total number of 1584 video loops. Patient flow chart and descriptive data with baseline characteristics are shown in Fig 1 and Table 1, respectively.

**Table 1:** Characteristics of the study population.

Characteristics of the study population	
Sex, male (%)	45 (68.2)
Birth weight (kg), mean (SD)	1.384 (1.02)
GA at birth (weeks), mean (SD)	28.9 (5.37)
Current weight (kg), mean (SD)	1.953 (920)
Current GA (weeks), mean (SD)	33.8 (4.23)
BP mean (mmHg), mean (SD)	53.3 (12.2)
HR (bpm), mean (SD)	153 (17.9)
SF ratio, mean (SD)	413.7 (62.0)
Antenatal steroids	
None (%)	19 (30.3)
Partial or complete (%)	46 (69.7)
Respiratory Support	
None (%)	22 (33.3)
HFNC (%)	30 (45.5)
nCPAP (%)	14 (21.2)
Surfactant applications	
None (%)	17 (25.75)
1 (%)	32 (48.5)
≥ 2 (%)	17 (25.75)
Inhaled Budesonide applications	
None (%)	50 (75.8)
≥ 1 (%)	16 (24.2)
State of the ductus arteriosus	
Closed (%)	55 (83.3)
Hemodynamically not significant (%)	3 (4.5)
Hemodynamically significant (%)	3 (4.5)
No echo (%)	5 (7.6)
APGAR at 5 minutes, median (IQR)	9 (8–9)

GA: Gestational age, BP: Blood pressure, HR: Heart rate, SF: Pulse oximetric saturation (SpO2)/Fraction of inspired oxygen (FiO2), HFNC: High flow nasal cannula, nCPAP: Nasal continuous positive airway pressure.

**Fig 2:**
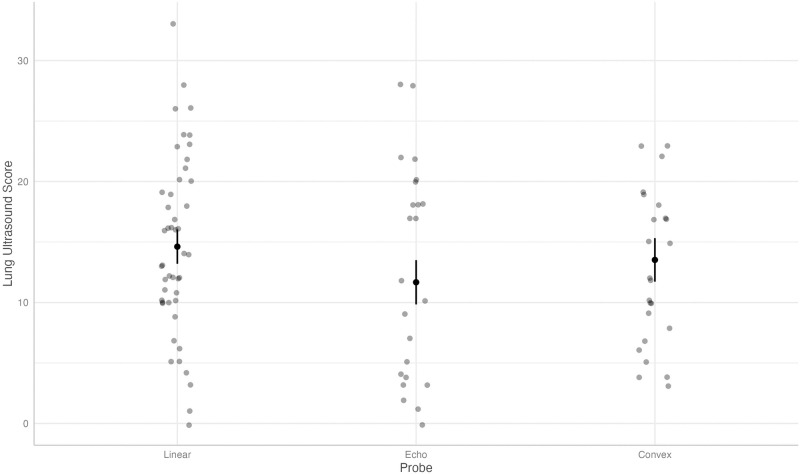
Predicted values (estimated marginal means) and 95% confidence intervals for lung ultrasound score grouped by type of ultrasound probe, according to the linear mixed-effects model.

Using a linear, mixed effects model, the results indicate that the NPLUS score is significantly lower when using the echo probe, with a predicted mean value of 11.67 (95% CI 9.84, 13.50) compared to the linear probe with a predicted mean value of 14.62 (95% CI 13.20, 16.04, coefficient -2.95, p < 0.001,). NPLUS score was slightly lower when using the microconvex probe compared to the linear probe, with a predicted mean value of 13.52 (95% CI 11.72, 15.32, coefficient -1.09, p = 0.19), depicted in Fig 2.

**Fig 3:**
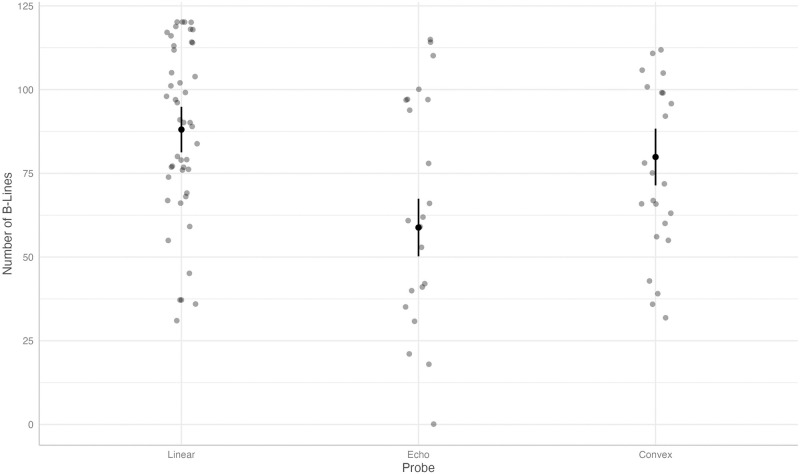
Predicted values (estimated marginal means) and 95% confidence intervals for the number of B-Lines, grouped by type of ultrasound probe, according to the linear mixed-effects model.

As a secondary outcome, we compared the total number of B-Lines. In this analysis, the predicted value for B-Lines using the linear probe was 88.07 (95% CI 81.26, 94.88) and the effects of the echo- and microconvex probes were statistically significant and negative with a predicted value of 58.82, (95% CI 50.22, 67.43, coefficient-29.25, p < .001) and 79.88, (95% CI 71.41, 88.36, coefficient -8.19, p = 0.03), respectively (see Fig 3). There was no significant impact of different evaluators.

**Fig 4:**
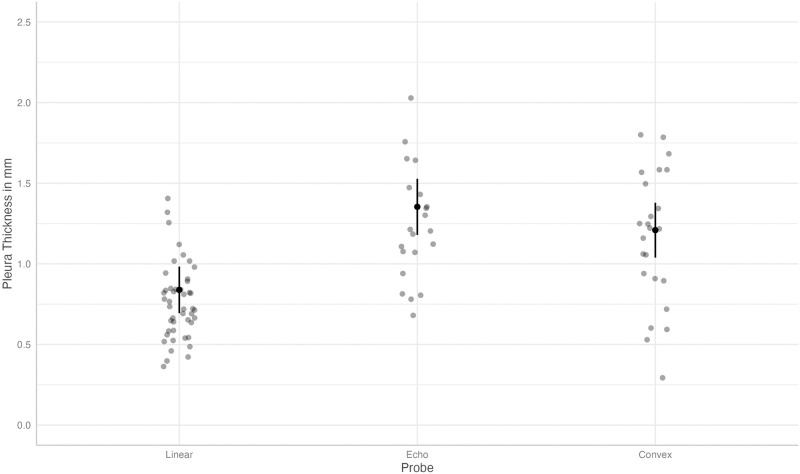
Predicted values (estimated marginal means) and 95% confidence intervals for pleura thickness, grouped by type of ultrasound probe, according to the linear mixed-effects model.

Predicted values for pleural line thickness using a linear probe was 0.84 mm (95% CI 0.69, 0.98). Pleural line thickness was higher with both echo- and microconvex probes with a predicted value of 1.35 (95% CI 1.18, 1.53, coefficients 0.51, p<0.01) and 1.21 (95% CI 1.04, 1.38, coefficient 0.37, p<0.01), respectively, as depicted in Fig 4. When further analyzing the pleural line thickness, we found that a higher APGAR score after 5 minutes (coefficient -0.31, p = 0.01) and higher SF ratio (coefficient -0.004, p = 0.02) was associated with a thinner pleural line. We also found significant differences between evaluators relating to pleural line thickness (coefficient 0.57, p = 0.03).

**Fig 5:**
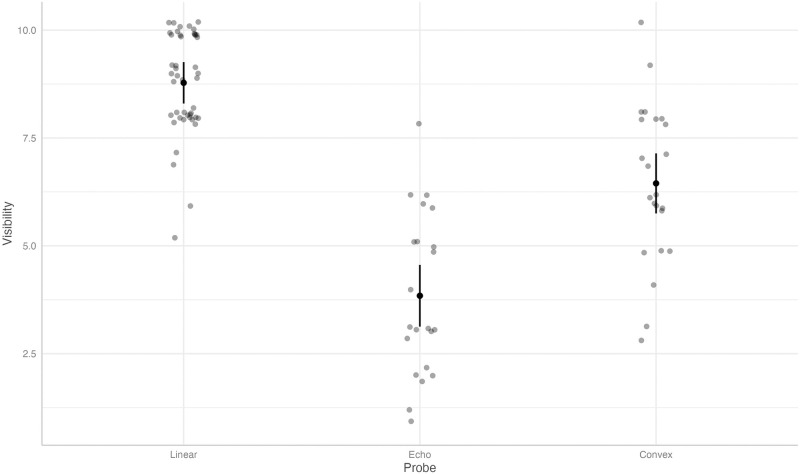
Predicted values (estimated marginal means) and 95% confidence intervals for the subjective visibility, grouped by type of ultrasound probe, according to the linear mixed-effects model.

The predicted value of the subjective visibility score was found to be 8.78 (95% CI 8.30, 9.26) with the linear probe, as opposed to 3.84 (95% CI 3.13, 4.56, coefficient -4.94, p<0.001) with the echo and 6.45 (95% CI 5.75, 7.14, coefficient -2.33, p<0.001) with the convex probe, see Fig 5.

In addition to the effect of the probes, we observed a particularly strong impact of the following clinical parameters on NPLUS score: Postnatal inhaled budesonide treatment showed a strong association with higher NPLUS score (coefficient 3.31, p = 0.03) whereas higher SF ratios were correlated with lower NPLUS score (coefficient -0.06, p = 0.001).

**Fig 6:**
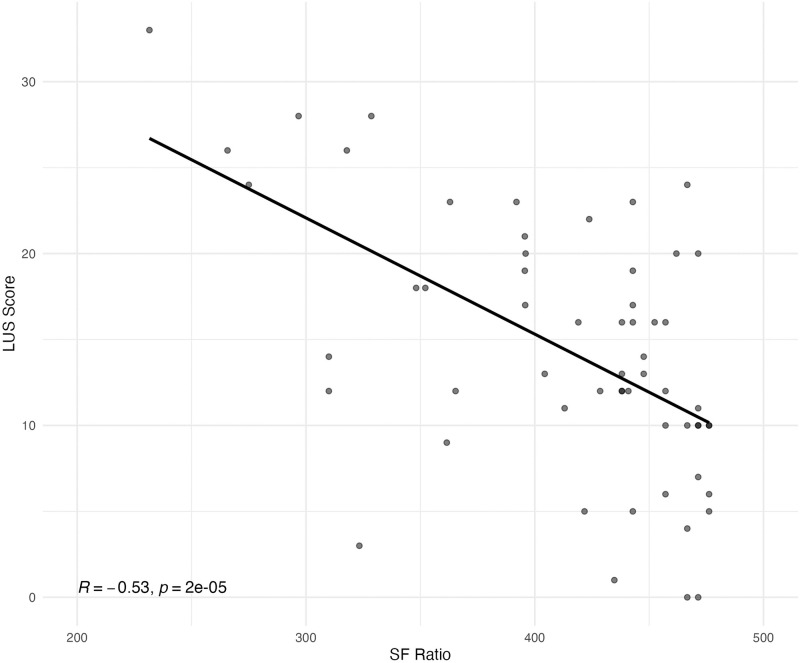
Spearman correlation between NPLUS Score and pulse oximetric saturation (SpO2)/Fraction of inspired oxygen (FiO2) (SF) ratio.

The Spearman correlation analysis revealed a moderate negative correlation between SF ratio and NPLUS score when using the linear probe (R = -0.53, p<0.001), as seen in Fig 6. We found a stronger correlation using the microconvex probe (R = -0.63, p<0.001) compared to the echo probe (R = -0.47, p = 0.011).

## Discussion

To our knowledge, this is the first study that compares the effect of different probes on lung ultrasound scores in neonates. We found significant differences between three types of ultrasound probes regarding lung ultrasound score, number of B-Lines, pleural line thickness, and subjective visibility.

Furthermore, we demonstrated a moderately strong correlation between NPLUS score and clinical factors, especially SF ratio. The study design allowed for both consistent level of expertise of the evaluators and ensured consistent machine settings.

Our findings suggest a significant difference in NPLUS score between different types of probes, with particularly lower scores when using an echo probe compared to a linear probe. Additionally, we demonstrated that experienced clinicians with high level of training in NPLUS rated visibility for assessment significantly lower when an echo probe was used. Due to the higher resolution and larger surface of the probe, more B-Lines are found when analyzing images generated by the linear probe. Subsequently, we found higher scores associated with this probe.

We believe that the inferior image quality, caused by the lower frequency that is linked to a lower superficial resolution generated by the echo probe, resulted in an underestimation of B-lines and consolidations and thus in lower scores.

In many of the analyses, we observed a relatively wide distribution of data points, especially in the NPLUS score (see Fig 2). However, we could not identify any significant, one-sided outliers that disrupted or influenced the analysis; rather, it represents a broad range of findings.

Although these findings essentially confirm the current recommendations, they are only partially congruent with previous research findings. In a study conducted by Bobbia et al., the authors evaluated the concordance of lung ultrasound and computed tomography (CT) scans using five different ultrasound probes in adult patients [28]. They found that the use of a cardiac or vascular probe improved diagnostic concordance. In contrast to that study, ours did not focus on diagnostic accuracy and did not include CT scans. The authors also found differences regarding the anatomical regions scanned in adults. We postulate that these differences are not expected in neonates, as adult patients tend to have more subcutaneous tissue and bones are ossified.

Recently, Schmickl et al. studied the impact of ultrasound settings and probes on the visibility of B-lines, using both in-vitro and in-vivo methods [29]. They found that machine settings influenced the visibility of B-lines, but there was no clear difference in the use of curvilinear and linear probes. In our study, we have predefined the machine settings to prevent any influence from variable adjustments, allowing the effect of the probe to be better highlighted.

In a study by Pivetta et al. in 2018, the sources of variability in the detection of B-lines were investigated using a prospective study of 96 clips from 50 adult patients [30]. The results were influenced by the type of probe used, the duration of the evaluation, and the level of expertise of the evaluator. In addition, the authors observed a significant reduction in B-lines when a microconvex probe was used. These results are therefore partly consistent with our data.

Gomond-Le Goff et al. conducted a study in 2020 to determine the diagnostic accuracy of LUS using different probes in neonates [31]. However, the study design was slightly different than ours, mainly because the authors introduced the “expertise-probe interaction factor”. We intentionally provided comparative data on probe utilization while controlling for experience variations. Furthermore, our study employed Brat et al.’s semi-quantitative score, in contrast to Gomond-Le Goff’s use of an adapted lung ultrasound score based on three patterns. It is interesting to note that in Gomond-Le Goff’s study, inexperienced raters had the lowest agreement when using non-linear probes. This could possibly correlate with the poorer visibility of the non-linear probes in our study, as this may pose a challenge for novices.

In a recent and well-executed study by Sett et al, it is highlighted that LUS findings using a linear probe in preterm infants are moderately correlated with oxygen saturation index and SF ratio (rho -0.63), which is consistent with our own findings [32]. Interestingly, the correlation between SF ratio and NPLUS score was better for the microconvex transducer than for the linear transducer in our study. This could suggest that while the linear transducer generates high-resolution and easily interpretable images, it may not provide the most accurate representation of the lung condition. However, our study was not primarily designed to investigate the relationship between LUS and lung condition and our study design did not allow for the inclusion of data on bronchopulmonary dysplasia and similar outcomes. While several studies analyzing this relationship have been published, we can only assess this matter to a limited extent [33–35].

Regarding the pleural line thickness, we can refer to previously published data by Alonso-Ojembarrena et al and found similar results [36]. Alonso suggests in her study that the pleural line thickness in healthy newborns should be less than 1mm. Although the interrater reliability was high in Alonso’s data, we found significant differences between evaluators, suggesting that even on expert levels, measuring this small, dynamic structure of less than 1mm might be challenging (see S1 Fig). Our data showed that in stable infants, a value of less than 1 mm was primarily measured when using the linear probe. In contrast, with the echo- and microconvex probe, mean values were generally more than 1mm.

Furthermore, we observed intriguing correlations between pleural thickness and the clinical condition—particularly SF ratio—which highlights the potential use of determining pleural thickness.

Finally, the relationship between NPLUS results and inhaled budesonide is interesting. In our study, neonates treated with inhaled budesonide appear to have a higher score. However, we explain this by the fact that these were infants who were typically above 40% of FiO2 over a period of several weeks, and thus these were more likely to be infants with chronic lung disease and a worse lung condition than the rest of the study population. In addition, only a relatively small number of infants received one or more doses of budesonide, which means that this finding should be interpreted with caution. In any case, further studies on this relationship are valuable and important.

## Limitations

There are several limitations of this study that should be noted. First, our sample was limited to a specific population of infants, and therefore the generalizability of our findings to other populations may be limited.

Furthermore, when it comes to the correlation of clinical parameters with NPLUS results, it must be mentioned that we analyzed exclusively hemodynamically stable infants with more than 1000g, with a mean SF ratio of 413, so we cannot provide data on this correlation regarding infants with severe RDS, especially considering that there is a certain overlap in SF ratio values in the context of mild RDS and normal lung function [37].

While our study employed rigorous quality control measures and trained clinicians, the potential for measurement error and inter-observer variability cannot be entirely excluded.

## Conclusion

The use of different types of ultrasound probes leads to varying results in the application of lung ultrasound in newborns, specifically regarding lung ultrasound score, number of B-Lines and pleural thickness, warranting a standardized selection of the probe for NPLUS.

While our results confirm the current recommendations of using a linear probe, our data show the possible extent of the differences between various ultrasound probes. The disparities we discovered call for caution in interpreting NPLUS findings, especially in the context of guiding therapies and communicating prognoses. Our study also suggests a strong correlation between NPLUS score and clinical parameters, validating the use of NPLUS and warranting further investigation in this area.

## Supporting information

S1 Fig Predicted values (estimated marginal means) for pleura thickness grouped by type of ultrasound probe and by evaluator, according to the linear mixed-effects model. (TIF)

S2 Fig Example image for linear probe. (TIF)

S3 Fig Example image for echo probe. (TIF)

S4 Fig Example image for convex probe. (TIF)

S1 Data Dataset including demographical data, clinical data, and ultrasound findings. (PDF)

S1 File Human subjects research checklist. (DOCX)
