# Supplementary figures and images for: Comparison of different types of ultrasound probes for lung ultrasound in neonates—A prospective randomized comparison study
Source: PLoS One. 2024 Jul 3;19(7):e0306472. doi: 10.1371/journal.pone.0306472 (PMC11221702; doi:10.1371/journal.pone.0306472)

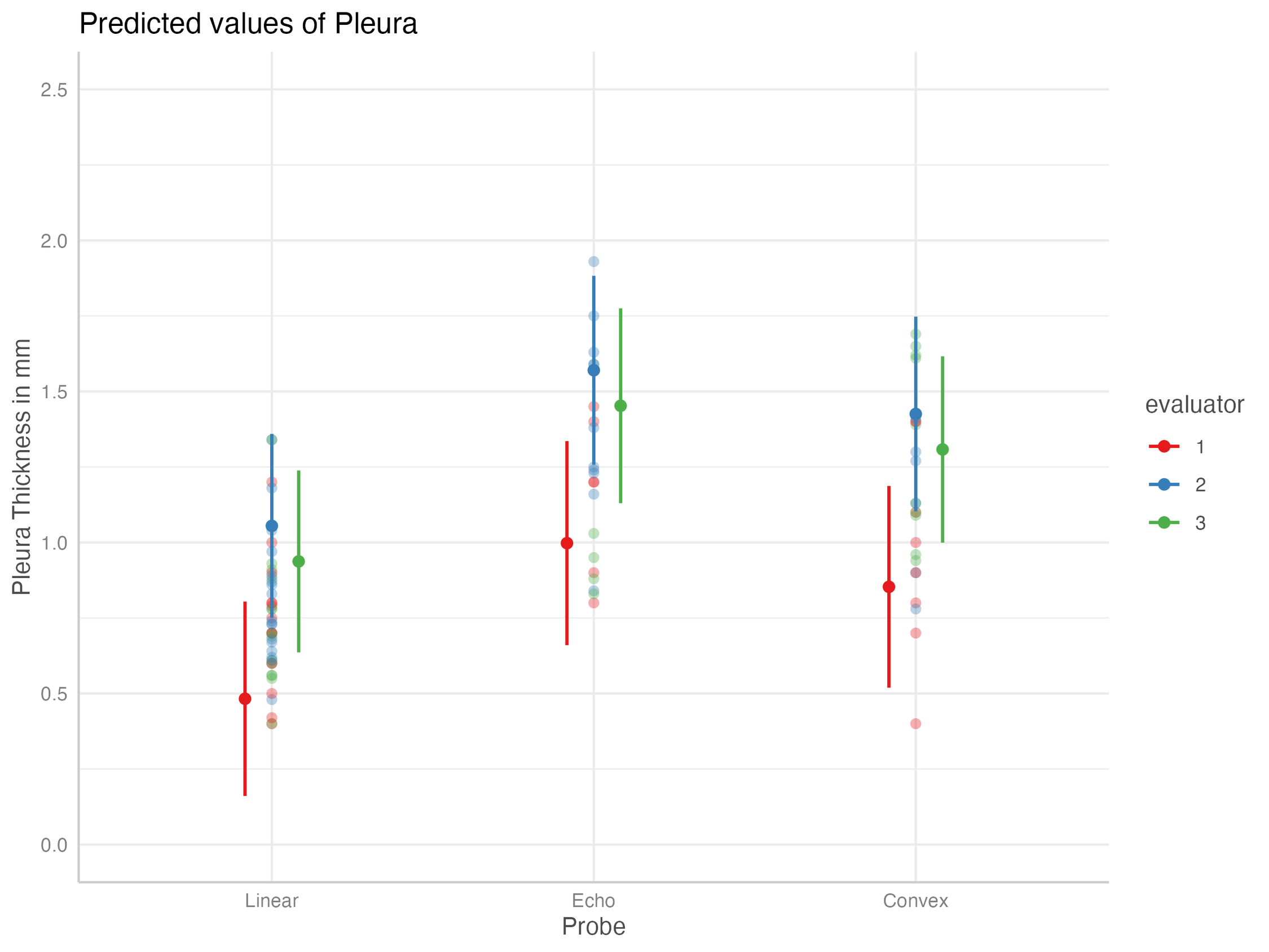

Supplement: S1 Fig — (TIF) [file pone.0306472.s001.tif]

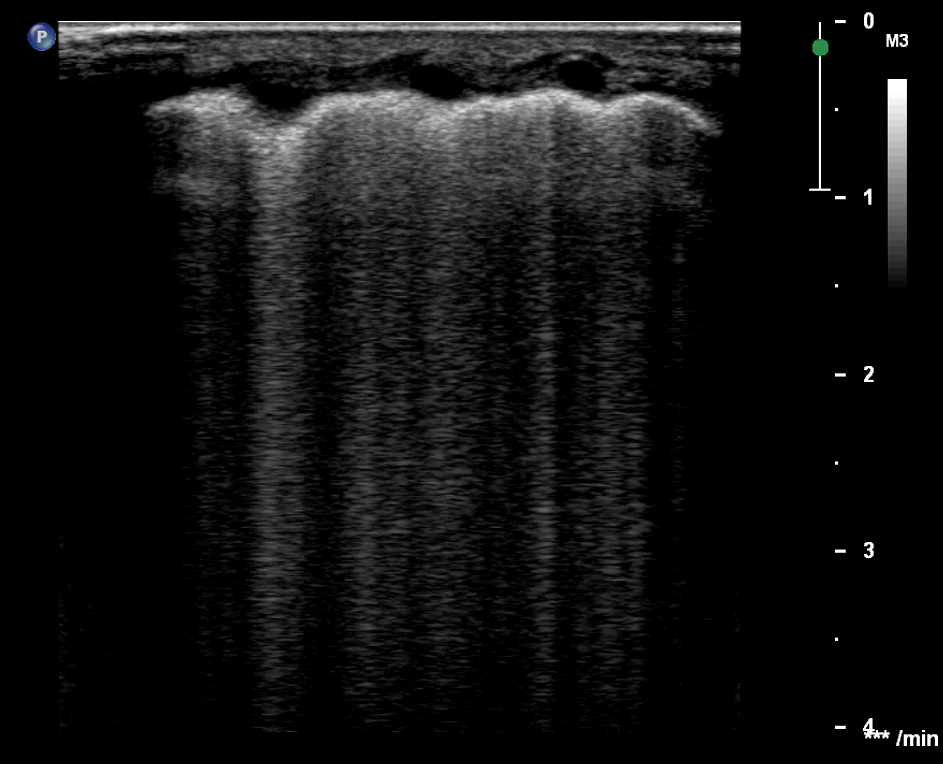

Supplement: S2 Fig — (TIF) [file pone.0306472.s002.tif]

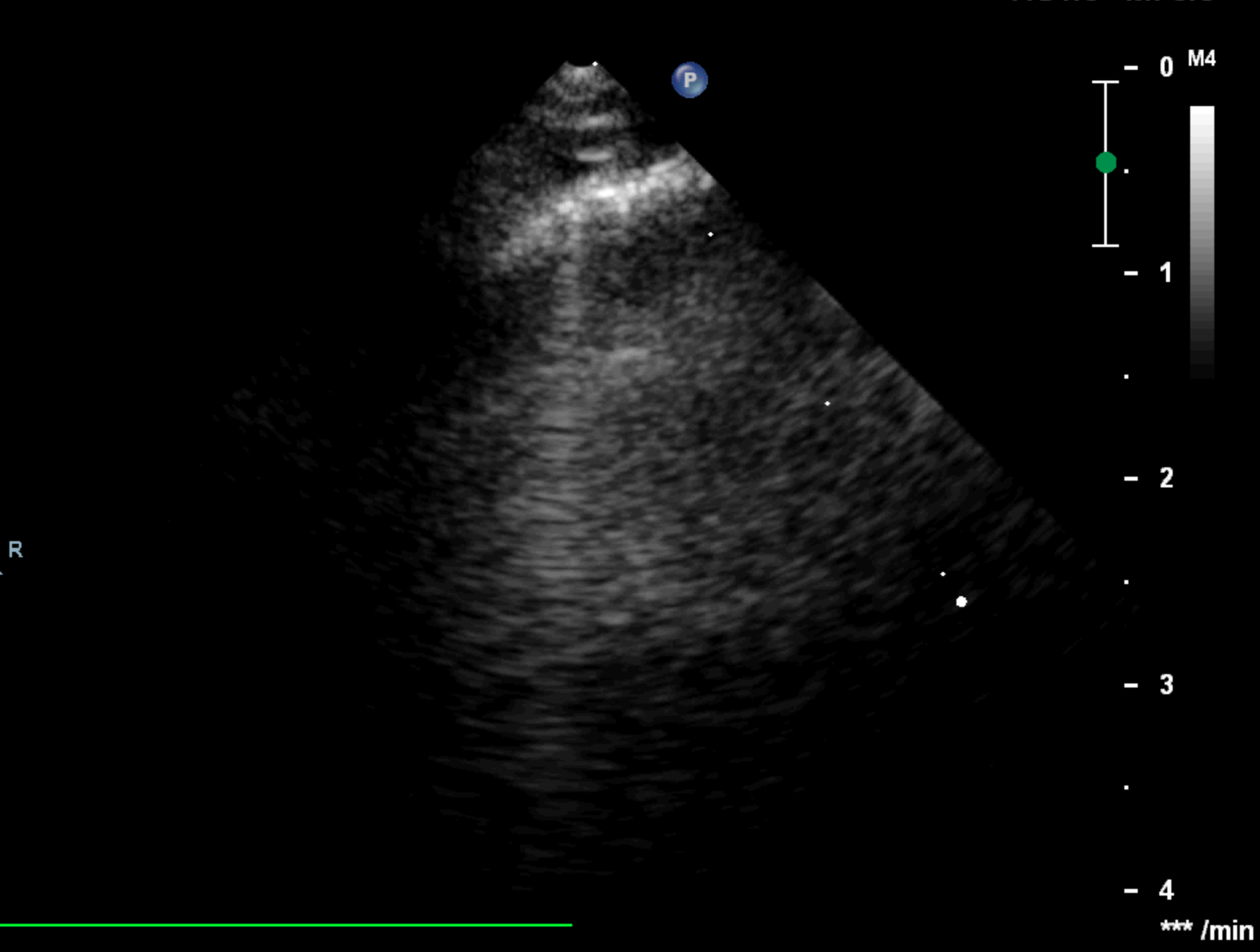

Supplement: S3 Fig — (TIF) [file pone.0306472.s003.tif]

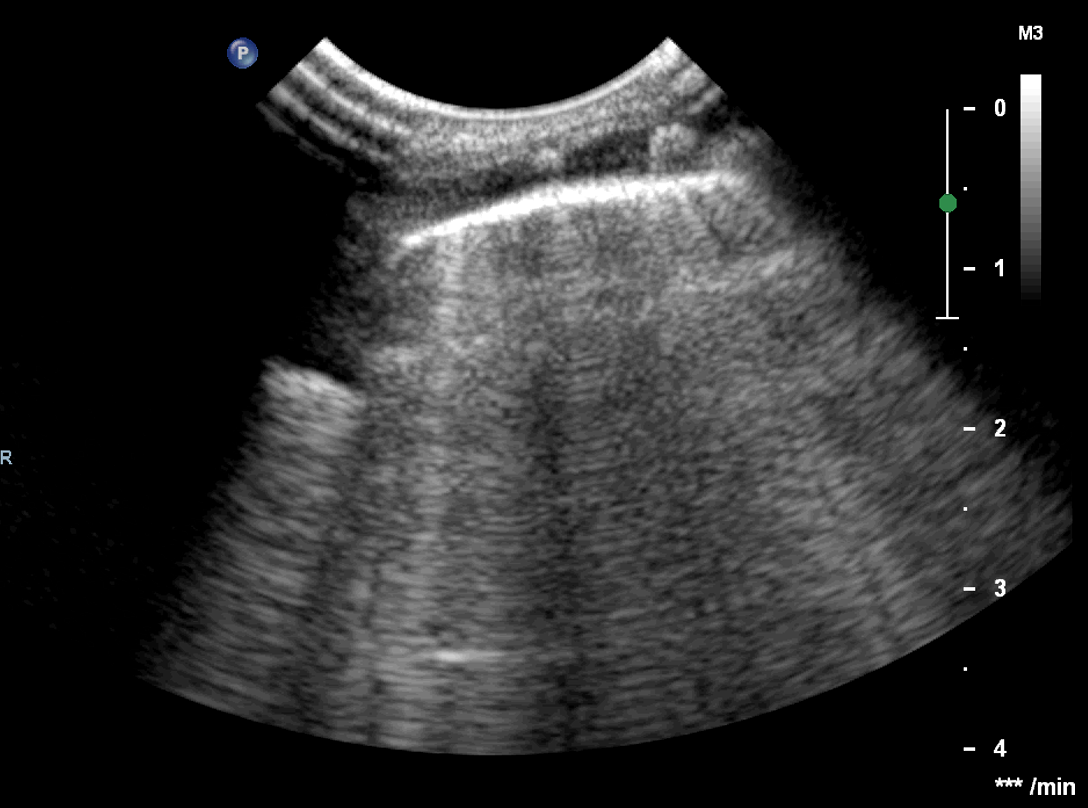

Supplement: S4 Fig — (TIF) [file pone.0306472.s004.tif]
