# Supplementary material for: Comparison of different types of ultrasound probes for lung ultrasound in neonates—A prospective randomized comparison study
Source: PLoS One. 2024 Jul 3;19(7):e0306472. doi: 10.1371/journal.pone.0306472 (PMC11221702; doi:10.1371/journal.pone.0306472)
Supplement: S1 Data — (PDF) [file pone.0306472.s005.pdf]

PID	altprobe	examiner	evaluator	ssw	ssw.d	dol	sex	bw	weight	fiO2	support	ant_steroid	surfactant	budesonid	hf	rr.mean	spo2	pda_akt	sepsis_akt	APGAR1	APGAR5	APGAR10	Probe	alines	blines
1,00	3	3	1	26,00	3,00	90,00	2	0,72	3,10	21,00	1	2	3	2,00	140,00	61,00	99,00	0	1	6,00	8,00	9,00	PR1	0,00	3,00
1,00	3	3	1	26,00	3,00	90,00	2	0,72	3,10	21,00	1	2	3	2,00	140,00	61,00	99,00	0	1	6,00	8,00	9,00	PR3	0,00	10,00
2,00	3	3	1	27,00	1,00	44,00	1	0,85	1,72	26,00	2	2	2	0,00	167,00	30,00	95,00	0	0	8,00	9,00	9,00	PR1	0,00	9,00
2,00	3	3	1	27,00	1,00	44,00	1	0,85	1,72	26,00	2	2	2	0,00	167,00	30,00	95,00	0	0	8,00	9,00	9,00	PR3	0,00	3,00
3,00	2	2	1	35,00	3,00	11,00	2	1,62	1,96		0	0	0	0,00	149,00			0	0	9,00	9,00	9,00	PR1	0,00	8,00
3,00	2	2	1	35,00	3,00	11,00	2	1,62	1,96		0	0	0	0,00	149,00			0	0	9,00	9,00	9,00	PR2	0,00	0,00
4,00	3	2	3	39,00	1,00	19,00	2	3,02	3,35		0	0	0	0,00		78,00	95,00	0	0	9,00	10,00	10,00	PR1	1,00	1,00
4,00	3	2	3	39,00	1,00	19,00	2	3,02	3,35		0	0	0	0,00		78,00	95,00	0	0	9,00	10,00	10,00	PR3	1,00	1,00
5,00	2	3	2	37,00	1,00	5,00	2	2,88	2,88	21,00	0	0	0	0,00	109,00	59,00	99,00		0	9,00	9,00	10,00	PR1	0,00	4,00
5,00	2	3	2	37,00	1,00	5,00	2	2,88	2,88	21,00	0	0	0	0,00	109,00	59,00	99,00		0	9,00	9,00	10,00	PR2	1,00	0,00
6,00	2	2	2	27,00	2,00	38,00	2	1,13	1,55	25,00	1	2	1	3,00		48,00	98,00	0	0	8,00	9,00	9,00	PR1	0,00	10,00
6,00	2	2	2	27,00	2,00	38,00	2	1,13	1,55	25,00	1	2	1	3,00		48,00	98,00	0	0	8,00	9,00	9,00	PR2	1,00	10,00
7,00	2	3	2	25,00	2,00	41,00	1	0,85	1,26	21,00	2	2	1	0,00	146,00	32,00	96,00	0	0	5,00	8,00	8,00	PR1	0,00	8,00
7,00	2	3	2	25,00	2,00	41,00	1	0,85	1,26	21,00	2	2	1	0,00	146,00	32,00	96,00	0	0	5,00	8,00	8,00	PR2	1,00	5,00
9,00	2	3	1	32,00	0,00	16,00	2	1,62	2,13	21,00	0	2	0	0,00	136,00		93,00		0	9,00	9,00	9,00	PR1	0,00	9,00
9,00	2	3	1	32,00	0,00	16,00	2	1,62	2,13	21,00	0	2	0	0,00	136,00		93,00		0	9,00	9,00	9,00	PR2	1,00	7,00
10,00	3	2	3	25,00	0,00	58,00	2	0,88	1,82	21,00	1	0	2	1,00	166,00	53,00	92,00	0	0	7,00	9,00	9,00	PR1	1,00	3,00
10,00	3	2	3	25,00	0,00	58,00	2	0,88	1,82	21,00	1	0	2	1,00	166,00	53,00	92,00	0	0	7,00	9,00	9,00	PR3	1,00	6,00
11,00	2	2	1	28,00	3,00	15,00	2	1,22	1,33	21,00	1	2	0	0,00	176,00	42,00	97,00	0	0				PR1	0,00	10,00
11,00	2	2	1	28,00	3,00	15,00	2	1,22	1,33	21,00	1	2	0	0,00	176,00	42,00	97,00	0	0				PR2	0,00	10,00
12,00	3	2	3	28,00	3,00	22,00	2	1,26	1,63	21,00	1	2	0	0,00	155,00	39,00		1	0	8,00	9,00	9,00	PR1	0,00	3,00
12,00	3	2	3	28,00	3,00	22,00	2	1,26	1,63	21,00	1	2	0	0,00	155,00	39,00		1	0	8,00	9,00	9,00	PR3	1,00	6,00
13,00	3	2	3	27,00	0,00	12,00	2	1,05	1,16	21,00	2	0	1	0,00	168,00	48,00	100,00	0	0				PR1	0,00	10,00
13,00	3	2	3	27,00	0,00	12,00	2	1,05	1,16	21,00	2	0	1	0,00	168,00	48,00	100,00	0	0				PR3	1,00	5,00
14,00	2	3	1	26,00	3,00	36,00	1	0,83	1,30	23,00	1	2	1	0,00	170,00	46,00	91,00	0	0	8,00	9,00	9,00	PR1	0,00	10,00
14,00	2	3	1	26,00	3,00	36,00	1	0,83	1,30	23,00	1	2	1	0,00	170,00	46,00	91,00	0	0	8,00	9,00	9,00	PR2	0,00	9,00
15,00	3	2	1	40,00	1,00	9,00	2	3,28	3,25	21,00	0	0	0	0,00	140,00	73,00	99,00	0	1	9,00	10,00	10,00	PR1	0,00	0,00
15,00	3	2	1	40,00	1,00	9,00	2	3,28	3,25	21,00	0	0	0	0,00	140,00	73,00	99,00	0	1	9,00	10,00	10,00	PR3	0,00	10,00
16,00	3	1	2	24,00	2,00	52,00	2	0,66	1,14	27,00	2	2	1		164,00	60,00	98,00	0	1	2,00	4,00	5,00	PR1	1,00	10,00
16,00	3	1	2	24,00	2,00	52,00	2	0,66	1,14	27,00	2	2	1		164,00	60,00	98,00	0	1	2,00	4,00	5,00	PR3	0,00	8,00
17,00	2	1	2	24,00	2,00	52,00	2	0,70	1,34	38,00	2	2	1	0,00	150,00	50,00	88,00	0	0	8,00	9,00	9,00	PR1	1,00	9,00
17,00	2	1	2	24,00	2,00	52,00	2	0,70	1,34	38,00	2	2	1	0,00	150,00	50,00	88,00	0	0	8,00	9,00	9,00	PR2	0,00	10,00
18,00	3	1	3	26,00	2,00	20,00	1	0,97	1,05	30,00	2	1	1		150,00	60,00	93,00	0	0	8,00	9,00	9,00	PR1	0,00	10,00
18,00	3	1	3	26,00	2,00	20,00	1	0,97	1,05	30,00	2	1	1		150,00	60,00	93,00	0	0	8,00	9,00	9,00	PR3	0,00	10,00
19,00	3	2	3	26,00	3,00	39,00	2	0,80	1,17	21,00	1	0	1	0,00	161,00	48,00	93,00	2	1	8,00	8,00	9,00	PR1	0,00	10,00
19,00	3	2	3	26,00	3,00	39,00	2	0,80	1,17	21,00	1	0	1	0,00	161,00	48,00	93,00	2	1	8,00	8,00	9,00	PR3	0,00	10,00
20,00	3	2	1	26,00	3,00	23,00	2	0,98	1,04	21,00	2	0	1	0,00	171,00	43,00	88,00	0	0	7,00	8,00	8,00	PR1	1,00	6,00
20,00	3	2	1	26,00	3,00	23,00	2	0,98	1,04	21,00	2	0	1	0,00	171,00	43,00	88,00	0	0	7,00	8,00	8,00	PR3	1,00	5,00
21,00	3	3	1	34,00	2,00	4,00	1	1,67	1,75	21,00	0	1	0	0,00	144,00	61,00	99,00	1	0	8,00	9,00	9,00	PR1	1,00	2,00
21,00	3	3	1	34,00	2,00	4,00	1	1,67	1,75	21,00	0	1	0	0,00	144,00	61,00	99,00	1	0	8,00	9,00	9,00	PR3	1,00	2,00
22,00	3	2	3	25,00	5,00	35,00	2	0,72	1,07	21,00	1	2	1	0,00	170,00	45,00	92,00	0	0	8,00	9,00	9,00	PR1	0,00	10,00
22,00	3	2	3	25,00	5,00	35,00	2	0,72	1,07	21,00	1	2	1	0,00	170,00	45,00	92,00	0	0	8,00	9,00	9,00	PR3	0,00	10,00
23,00	2	3	2	27,00	0,00	38,00	2	1,00	1,63	21,00	1	0	2	0,00	160,00	57,00	92,00	0	0	7,00	9,00	9,00	PR1	1,00	10,00
23,00	2	3	2	27,00	0,00	38,00	2	1,00	1,63	21,00	1	0	2	0,00	160,00	57,00	92,00	0	0	7,00	9,00	9,00	PR2	1,00	1,00
24,00	3	3	2	27,00	5,00	9,00	1	1,03	1,17	21,00	2	1	1	0,00	144,00	42,00	92,00	0	0	9,00	9,00	10,00	PR1	0,00	10,00
24,00	3	3	2	27,00	5,00	9,00	1	1,03	1,17	21,00	2	1	1	0,00	144,00	42,00	92,00	0	0	9,00	9,00	10,00	PR3	0,00	10,00
25,00	3	2	3	24,00	4,00	124,00	2	0,48	3,09	21,00	0	1	1	0,00	138,00			0	1	7,00	8,00	9,00	PR1	0,00	0,00
25,00	3	2	3	24,00	4,00	124,00	2	0,48	3,09	21,00	0	1	1	0,00	138,00			0	1	7,00	8,00	9,00	PR3	1,00	2,00
26,00	3	1	3	23,00	4,00	90,00	1	0,57	1,98	28,00	1	2	3	2,00	130,00	52,00	92,00	0	0	8,00	9,00	9,00	PR1	1,00	4,00
26,00	3	1	3	23,00	4,00	90,00	1	0,57	1,98	28,00	1	2	3	2,00	130,00	52,00	92,00	0	0	8,00	9,00	9,00	PR3	1,00	8,00
27,00	2	2	1	25,00	1,00	43,00	2	0,65	1,29	23,00	1	2	2	1,00	159,00	47,00	91,00	0	1	7,00	9,00	9,00	PR1	0,00	10,00
27,00	2	2	1	25,00	1,00	43,00	2	0,65	1,29	23,00	1	2	2	1,00	159,00	47,00	91,00	0	1	7,00	9,00	9,00	PR2	0,00	10,00
28,00	3	2	3	39,00	6,00	16,00	2	3,95	3,91	21,00	0	0	0		138,00		93,00	0	0	9,00	10,00	10,00	PR1	1,00	6,00
28,00	3	2	3	39,00	6,00	16,00	2	3,95	3,91	21,00	0	0	0		138,00		93,00	0	0	9,00	10,00	10,00	PR3	1,00	4,00
29,00	3	3	2	37,00	3,00	2,00	1	3,03	3,03	21,00	0	0	0	0,00	129,00				0	9,00	10,00	10,00	PR1	1,00	5,00
29,00	3	3	2	37,00	3,00	2,00	1	3,03	3,03	21,00	0	0	0	0,00	129,00				0	9,00	10,00	10,00	PR3	1,00	5,00
30,00	2	2	3	27,00	6,00	5,00	2	1,27	1,22	21,00	1	2	1	0,00	145,00	47,00	100,00	1	0	7,00	9,00	9,00	PR1	1,00	10,00
30,00	2	2	3	27,00	6,00	5,00	2	1,27	1,22	21,00	1	2	1	0,00	145,00	47,00	100,00	1	0	7,00	9,00	9,00	PR2	1,00	3,00
31,00	2	3	2	41,00	3,00	7,00	1	3,78	3,97	23,00	1	0	1	0,00	126,00	76,00	97,00	0	0	5,00	6,00	7,00	PR1	1,00	9,00
31,00	2	3	2	41,00	3,00	7,00	1	3,78	3,97	23,00	1	0	1	0,00	126,00	76,00	97,00	0	0	5,00	6,00	7,00	PR2	1,00	2,00
32,00	3	2	3	25,00	6,00	63,00	2	0,58	1,86	21,00	1	2	2	1,00	130,00	46,00	89,00	0	0	7,00	9,00	9,00	PR1	0,00	10,00
32,00	3	2	3	25,00	6,00	63,00	2	0,58	1,86	21,00	1	2	2	1,00	130,00	46,00	89,00	0	0	7,00	9,00	9,00	PR3	1,00	9,00
33,00	3	3	2	28,00	1,00	18,00	2	1,39	1,50	23,00	1	2	1	0,00	166,00	71,00	95,00	0	0	8,00	9,00	9,00	PR1	1,00	4,00

33,00	3	3	2	28,00	1,00	18,00	2	1,39	1,50	23,00	1	2	1	0,00	166,00	71,00	95,00	0	0	8,00	9,00	9,00	PR3	1,00	5,00
34,00	3	2	1	27,00	6,00	20,00	2	1,44	1,62	21,00	1	2	1	0,00	180,00	47,00	93,00	0	0	3,00	7,00	7,00	PR1	0,00	8,00
34,00	3	2	1	27,00	6,00	20,00	2	1,44	1,62	21,00	1	2	1	0,00	180,00	47,00	93,00	0	0	3,00	7,00	7,00	PR3	1,00	5,00
35,00	2	3	1	24,00	0,00	95,00	2	0,55	2,36	21,00	0	2	1	0,00	150,00	68,00	99,00	0	0	6,00	7,00	8,00	PR1	0,00	10,00
35,00	2	3	1	24,00	0,00	95,00	2	0,55	2,36	21,00	0	2	1	0,00	150,00	68,00	99,00	0	0	6,00	7,00	8,00	PR2	1,00	3,00
36,00	3	3	1	24,00	0,00	86,00	1	0,58	2,24	21,00	0	2	1	0,00	177,00	65,00	90,00	0	0	5,00	7,00	9,00	PR1	0,00	8,00
36,00	3	3	1	24,00	0,00	86,00	1	0,58	2,24	21,00	0	2	1	0,00	177,00	65,00	90,00	0	0	5,00	7,00	9,00	PR3	0,00	8,00
37,00	3	2	1	41,00	3,00	3,00	1	3,15	3,15	21,00	0	0	0	0,00	128,00	59,00	99,00	0	0	5,00	7,00	9,00	PR1	1,00	8,00
37,00	3	2	1	41,00	3,00	3,00	1	3,15	3,15	21,00	0	0	0	0,00	128,00	59,00	99,00	0	0	5,00	7,00	9,00	PR3	1,00	6,00
38,00	2	2	3	26,00	2,00	24,00	2	0,92	1,10	35,00	2	2	3	2,00	177,00	40,00	93,00	0	1	7,00	8,00	9,00	PR1	0,00	10,00
38,00	2	2	3	26,00	2,00	24,00	2	0,92	1,10	35,00	2	2	3	2,00	177,00	40,00	93,00	0	1	7,00	8,00	9,00	PR2	0,00	10,00
39,00	2	2	1	40,00	0,00	16,00	2	4,35	4,43	30,00	1	0	0	0,00	141,00	72,00	97,00	0	1	6,00	8,00	8,00	PR1	1,00	5,00
39,00	2	2	1	40,00	0,00	16,00	2	4,35	4,43	30,00	1	0	0	0,00	141,00	72,00	97,00	0	1	6,00	8,00	8,00	PR2	0,00	
40,00	2	2	3	26,00	2,00	27,00	1	0,88	1,20	21,00	1	1	1	0,00	174,00	58,00	93,00	2	0	8,00	9,00	9,00	PR1	0,00	10,00
40,00	2	2	3	26,00	2,00	27,00	1	0,88	1,20	21,00	1	1	1	0,00	174,00	58,00	93,00	2	0	8,00	9,00	9,00	PR2	0,00	10,00
41,00	2	3	2	26,00	2,00	25,00	1	0,86	1,06	24,00	1	1	1	0,00	177,00	47,00	95,00	0	0	8,00	9,00	9,00	PR1	0,00	10,00
41,00	2	3	2	26,00	2,00	25,00	1	0,86	1,06	24,00	1	1	1	0,00	177,00	47,00	95,00	0	0	8,00	9,00	9,00	PR2	1,00	4,00
42,00	2	2	1	27,00	2,00	23,00	1	0,91	1,25	23,00	2	0	2	1,00	175,00	45,00	81,00	0	0	7,00	9,00	9,00	PR1	0,00	10,00
42,00	2	2	1	27,00	2,00	23,00	1	0,91	1,25	23,00	2	0	2	1,00	175,00	45,00	81,00	0	0	7,00	9,00	9,00	PR2	0,00	10,00
43,00	3	2	3	34,00	2,00	18,00	1	1,99	1,91	21,00	0	1	0	0,00	134,00	44,00	99,00	0	1	9,00	9,00	10,00	PR1	1,00	4,00
43,00	3	2	3	34,00	2,00	18,00	1	1,99	1,91	21,00	0	1	0	0,00	134,00	44,00	99,00	0	1	9,00	9,00	10,00	PR3	1,00	5,00
44,00	2	2	3	27,00	0,00	53,00	1	0,75	1,38	25,00	1	0	3	2,00	154,00	44,00		0	1	7,00	9,00	9,00	PR1	1,00	10,00
44,00	2	2	3	27,00	0,00	53,00	1	0,75	1,38	25,00	1	0	3	2,00	154,00	44,00		0	1	7,00	9,00	9,00	PR2	0,00	7,00
45,00	2	2	1	24,00	0,00	83,00	1	0,44	1,50	31,00	2	2	3	3,00		67,00	92,00	0		8,00	9,00	9,00	PR1	0,00	10,00
45,00	2	2	1	24,00	0,00	83,00	1	0,44	1,50	31,00	2	2	3	3,00		67,00	92,00	0		8,00	9,00	9,00	PR2	0,00	10,00
46,00	2	2	1	25,00	1,00	33,00	2	0,77	1,05	28,00	1	2	2	1,00	167,00	37,00	89,00	0	0	8,00	9,00	9,00	PR1	0,00	10,00
46,00	2	2	1	25,00	1,00	33,00	2	0,77	1,05	28,00	1	2	2	1,00	167,00	37,00	89,00	0	0	8,00	9,00	9,00	PR2	1,00	
47,00	3	2	3	26,00	6,00	36,00	2	0,90	1,24	21,00	2	2	1	0,00	192,00	39,00	98,00	0	0	7,00	9,00	9,00	PR1	1,00	5,00
47,00	3	2	3	26,00	6,00	36,00	2	0,90	1,24	21,00	2	2	1	0,00	192,00	39,00	98,00	0	0	7,00	9,00	9,00	PR3	1,00	8,00
48,00	3	2	3	27,00	6,00	54,00	2	0,85	2,21	21,00	0	1	3	2,00	151,00	60,00	100,00	0	1	8,00	9,00	9,00	PR1	1,00	7,00
48,00	3	2	3	27,00	6,00	54,00	2	0,85	2,21	21,00	0	1	3	2,00	151,00	60,00	100,00	0	1	8,00	9,00	9,00	PR3	1,00	10,00
49,00	2	2	3	27,00	6,00	54,00	2	0,99	2,22	21,00	0	1	3	2,00	149,00	53,00	100,00	0	1	8,00	9,00	9,00	PR1	1,00	5,00
49,00	2	2	3	27,00	6,00	54,00	2	0,99	2,22	21,00	0	1	3	2,00	149,00	53,00	100,00	0	1	8,00	9,00	9,00	PR2	1,00	4,00
50,00	2	2	1	23,00	0,00	116,00	2	0,58	3,11	21,00	0	2	2	1,00	150,00	75,00	94,00	0	1	5,00	7,00	9,00	PR1	1,00	3,00
50,00	2	2	1	23,00	0,00	116,00	2	0,58	3,11	21,00	0	2	2	1,00	150,00	75,00	94,00	0	1	5,00	7,00	9,00	PR2	0,00	
54,00	3	2	3	28,00	0,00	57,00	2	1,15	2,20	21,00	0	2	0	0,00	140,00	65,00	98,00	0	0	8,00	9,00	9,00	PR1	1,00	4,00
54,00	3	2	3	28,00	0,00	57,00	2	1,15	2,20	21,00	0	2	0	0,00	140,00	65,00	98,00	0	0	8,00	9,00	9,00	PR3	1,00	4,00
58,00	3	3	2	26,00	0,00	86,00	2	0,82	2,75	21,00	0	1	1	0,00	116,00	67,00	95,00	0	0	8,00	9,00	9,00	PR1	1,00	8,00
58,00	3	3	2	26,00	0,00	86,00	2	0,82	2,75	21,00	0	1	1	0,00	116,00	67,00	95,00	0	0	8,00	9,00	9,00	PR3	1,00	5,00
59,00	3	3	2	24,00	3,00	43,00	2	0,70	1,17	36,00	2	1	2	1,00	172,00	44,00	99,00	0	0	8,00	9,00	9,00	PR1	0,00	10,00
59,00	3	3	2	24,00	3,00	43,00	2	0,70	1,17	36,00	2	1	2	1,00	172,00	44,00	99,00	0	0	8,00	9,00	9,00	PR3	1,00	9,00
60,00	3	2	3	30,00	1,00	7,00	1	1,61	1,79	27,00	2	2	1	0,00	155,00	38,00	94,00	0	1	9,00	9,00	9,00	PR1	0,00	10,00
60,00	3	2	3	30,00	1,00	7,00	1	1,61	1,79	27,00	2	2	1	0,00	155,00	38,00	94,00	0	1	9,00	9,00	9,00	PR3	1,00	5,00
62,00	2	3	2	27,00	4,00	67,00	2	0,93	2,42	21,00	0	2	1	0,00	156,00	69,00	94,00	0	0	8,00	9,00	9,00	PR1	1,00	10,00
62,00	2	3	2	27,00	4,00	67,00	2	0,93	2,42	21,00	0	2	1	0,00	156,00	69,00	94,00	0	0	8,00	9,00	9,00	PR2	0,00	5,00
63,00	2	3	2	41,00	4,00	5,00	2	4,00	4,15	26,00	1	0	1	0,00	127,00	57,00	94,00	0	1	5,00	6,00	6,00	PR1	1,00	10,00
63,00	2	3	2	41,00	4,00	5,00	2	4,00	4,15	26,00	1	0	1	0,00	127,00	57,00	94,00	0	1	5,00	6,00	6,00	PR2	0,00	7,00
64,00	2	2	3	29,00	2,00	7,00	1	1,08	1,04	23,00	1	1	1	0,00	156,00	47,00	100,00	0	1	8,00	8,00	9,00	PR1	1,00	4,00
64,00	2	2	3	29,00	2,00	7,00	1	1,08	1,04	23,00	1	1	1	0,00	156,00	47,00	100,00	0	1	8,00	8,00	9,00	PR2	1,00	2,00
65,00	3	3	2	25,00	4,00	47,00	2	0,85	1,20	25,00	1	2	1	0,00	152,00	57,00	99,00	0	0	8,00	8,00	9,00	PR1	0,00	9,00
65,00	3	3	2	25,00	4,00	47,00	2	0,85	1,20	25,00	1	2	1	0,00	152,00	57,00	99,00	0	0	8,00	8,00	9,00	PR3	0,00	8,00
66,00	2	1	3	25,00	2,00	47,00	2	0,80	1,71	23,00	1		1	0,00	162,00	60,00	93,00	0	1	8,00	9,00	9,00	PR1	1,00	6,00
66,00	2	1	3	25,00	2,00	47,00	2	0,80	1,71	23,00	1		1	0,00	162,00	60,00	93,00	0	1	8,00	9,00	9,00	PR2	1,00	5,00
68,00	2	3	2	30,00	4,00	10,00	2	1,40	1,39	21,00	0	2	1	0,00	146,00	44,00	96,00	0	0	8,00	9,00	9,00	PR1	1,00	8,00
68,00	2	3	2	30,00	4,00	10,00	2	1,40	1,39	21,00	0	2	1	0,00	146,00	44,00	96,00	0	0	8,00	9,00	9,00	PR2	2,00	4,00
69,00	2	3	2	27,00	1,00	53,00	1	0,76	1,67	21,00	1	2	2	1,00	142,00	38,00	98,00	0	0	8,00	9,00	9,00	PR1	0,00	10,00
69,00	2	3	2	27,00	1,00	53,00	1	0,76	1,67	21,00	1	2	2	1,00	142,00	38,00	98,00	0	0	8,00	9,00	9,00	PR2	0,00	10,00
70,00	2	3	2	34,00	5,00	4,00	2	2,31	2,25	21,00	1	0	0	0,00	140,00	70,00	98,00		0	8,00	8,00	9,00	PR1	2,00	2,00
70,00	2	3	2	34,00	5,00	4,00	2	2,31	2,25	21,00	1	0	0	0,00	140,00	70,00	98,00		0	8,00	8,00	9,00	PR2	2,00	7,00
71,00	3	3	2	27,00	3,00	30,00	2	1,01	1,25	30,00	1	2	1	0,00	155,00	59,00	93,00	0	0	8,00	9,00	9,00	PR1	1,00	10,00
71,00	3	3	2	27,00	3,00	30,00	2	1,01	1,25	30,00	1	2	1	0,00	155,00	59,00	93,00	0	0	8,00	9,00	9,00	PR3	0,00	10,00
72,00	2	3	2	29,00	4,00	5,00	1	1,02	1,03	22,00	1	2	1	0,00	158,00	36,00	97,00	2	0	8,00	9,00	9,00	PR1	1,00	10,00
72,00	2	3	2	29,00	4,00	5,00	1	1,02	1,03	22,00	1	2	1	0,00	158,00	36,00	97,00	2	0	8,00	9,00	9,00	PR2	1,00	4,00

blines_sum	ines_ICR_m	blines_rand	kons	lusscore	sscore.gesar	Pleura	visibility
55,00	2,00	4,00	0,00	1,00	11,00	0,80	8,00
39,00	5,00	3,00	0,00	2,00	10,00	1,40	3,00
69,00	3,00	4,00	0,00	1,00	12,00	0,40	9,00
55,00	2,00		0,00	1,00	12,00	0,40	7,00
79,00	3,00	7,00	0,00	1,00	14,00	1,00	7,00
19,00	0,00	2,00	0,00	0,00	9,00	2,00	2,00
24,00	1,00	3,00	0,00	0,00	5,00	0,70	7,00
	1,00	0,00	0,00	0,00	4,00	1,50	7,00
67,00	3,00	2,00	0,00	1,00	10,00	0,50	6,00
9,00	0,00	0,00	0,00	0,00	1,00	1,00	2,00
118,00	5,00	10,00	0,00	2,00	23,00	0,97	8,00
97,00	3,00	10,00	0,00	1,00	20,00	1,59	3,00
97,00	3,00	9,00	0,00	1,00	16,00	0,48	8,00
62,00	3,00	3,00	0,00	1,00	10,00	1,25	5,00
105,00	3,00	10,00	0,00	1,00	17,00	0,80	6,00
85,00	3,00	10,00	0,00	1,00	17,00	2,00	3,00
77,00	1,00	10,00	0,00	0,00	13,00	0,78	10,00
78,00	3,00	2,00	0,00	1,00	15,00	1,09	8,00
116,00	5,00	10,00	0,00	2,00	20,00	0,60	8,00
80,00	4,00	4,00	0,00	2,00	17,00	2,00	4,00
89,00	2,00	2,00	0,00	0,00	18,00	0,78	8,00
80,00	4,00	6,00	0,00	1,00	17,00	1,09	9,00
59,00	5,00	10,00	0,00	2,00	6,00	0,79	8,00
55,00	3,00	5,00	0,00	1,00	8,00	1,38	7,00
113,00	5,00	10,00	0,00	2,00	19,00	0,60	9,00
94,00	4,00	9,00	0,00	2,00	18,00	1,40	5,00
39,00	0,00	4,00	0,00	0,00	10,00	1,00	9,00
56,00	3,00	0,00	0,00	2,00	13,00	1,50	5,00
119,00	4,00	9,00	0,00	1,00	23,00	0,74	9,00
105,00	2,00	6,00	0,00	0,00	12,00	1,78	4,00
119,00	3,00	10,00	0,00	1,00	33,00	1,34	10,00
115,00	5,00	10,00	0,00	1,00	28,00	1,63	3,00
102,00	5,00	10,00	0,00	1,00	12,00	0,74	8,00
55,00	5,00	2,00	0,00	2,00	7,00	1,21	6,00
105,00	5,00	10,00	1,00	3,00	23,00	1,34	9,00
105,00	5,00	10,00	1,00	3,00	22,00	1,61	8,00
79,00	3,00	5,00	0,00	1,00	16,00	0,70	8,00
66,00	3,00	10,00	0,00	1,00	12,00	1,10	6,00
31,00	1,00	4,00	0,00	0,00	0,00	0,70	9,00
32,00	1,00	2,00	0,00	0,00	4,00	0,80	7,00
96,00	5,00	10,00	0,00	2,00	16,00	0,91	5,00
101,00	5,00	9,00	0,00	2,00	17,00	1,65	6,00
112,00	3,00	10,00	0,00	1,00	12,00	0,64	9,00
31,00	1,00	3,00	0,00	0,00	0,00	1,75	2,00
120,00	3,00	10,00	0,00	1,00	12,00	0,86	10,00
92,00	5,00	7,00	0,00	2,00	15,00	1,27	8,00
34,00	0,00	3,00	0,00	0,00	1,00	0,79	8,00
38,00	1,00	4,00	0,00	0,00	2,00	1,31	8,00
99,00	3,00	4,00	0,00	1,00	28,00	0,88	10,00
99,00	4,00	8,00	0,00	1,00	23,00	1,39	6,00
114,00	5,00	8,00	0,00	2,00	21,00	0,70	8,00
110,00	5,00	8,00	0,00	2,00	20,00	0,90	
48,00	3,00	5,00	0,00	1,00	5,00	0,97	10,00
43,00	3,00	1,00	0,00	1,00	4,00	1,39	10,00
73,00	3,00	9,00	0,00	1,00	11,00	0,51	7,00
65,00	3,00	5,00	0,00	1,00	9,00	1,88	8,00
74,00	3,00	10,00	0,00	1,00	10,00	0,69	8,00
59,00	2,00	3,00	0,00	0,00	9,00	0,95	4,00
66,00	2,00	7,00	0,00	0,00	5,00	0,67	8,00
42,00	1,00	2,00	0,00	0,00	4,00	0,84	8,00
117,00	5,00	10,00	0,00	2,00	22,00	0,61	10,00
106,00	3,00	8,00	0,00	1,00	18,00	0,94	7,00
76,00	2,00	4,00	0,00	0,00	11,00	0,83	8,00

72,00	2,00	2,00	0,00	0,00	10,00	0,90	5,00
84,00	3,00	8,00	0,00	2,00	16,00	1,20	8,00
43,00	2,00	5,00	0,00	1,00	6,00	0,90	5,00
79,00	5,00	4,00	0,00	2,00	20,00	0,90	8,00
61,00	2,00	3,00	0,00	1,00	18,00		6,00
67,00	5,00	5,00	0,00	2,00	12,00	0,60	8,00
66,00	3,00	4,00	0,00	2,00	17,00	1,40	5,00
68,00	2,00	4,00	0,00	1,00	10,00	0,50	8,00
63,00	3,00	4,00	0,00	1,00	8,00	0,70	4,00
120,00	5,00	10,00	1,00	3,00	26,00	4,20	10,00
114,00	5,00	10,00	0,00	2,00	28,00	4,75	3,00
36,00	3,00	5,00	0,00	1,00	3,00	1,00	9,00
	3,00		0,00	1,00	3,00	1,20	6,00
118,00	5,00	10,00	0,00	2,00	19,00	0,40	9,00
97,00	5,00	5,00	0,00	2,00	18,00	0,88	5,00
120,00	3,00	10,00	0,00	1,00	17,00	1,18	7,00
66,00	2,00	5,00	0,00	0,00	12,00	1,24	3,00
98,00	5,00	10,00	0,00	2,00	18,00	0,75	8,00
97,00			0,00	2,00	17,00	1,45	3,00
59,00	2,00	2,00	0,00	0,00	7,00	0,55	6,00
56,00	3,00	3,00	0,00	1,00	7,00	1,10	6,00
120,00	5,00		0,00	1,00	21,00	0,56	10,00
112,00	3,00		0,00	1,00	21,00	1,34	6,00
120,00	5,00	10,00	1,00	3,00	28,00	2,30	10,00
120,00	5,00	10,00	0,00	2,00	28,00	1,40	4,00
114,00	5,00	10,00	0,00	2,00	26,00	0,80	8,00
100,00		10,00	0,00	1,00	22,00	0,80	3,00
80,00	3,00		0,00	1,00	10,00	0,60	10,00
75,00	4,00		0,00	1,00	10,00	0,96	6,00
77,00	3,00		0,00	1,00	10,00	0,56	8,00
67,00	5,00		0,00	2,00	9,00	1,13	6,00
45,00	3,00		0,00	1,00	5,00	0,56	9,00
35,00	2,00		0,00	0,00	3,00	1,03	5,00
77,00	1,00		0,00	0,00	13,00	0,42	
0,00			0,00	2,00	22,00	1,20	1,00
37,00	2,00	6,00	0,00	1,00	4,00	0,70	10,00
36,00	2,00	2,00	0,00	0,00	3,00	1,69	8,00
89,00	4,00	7,00	0,00	1,00	16,00	0,73	9,00
60,00	1,00	5,00	0,00	0,00	4,00	1,13	8,00
116,00	5,00	10,00	0,00	1,00	24,00	0,68	10,00
112,00	5,00	8,00	0,00	1,00	23,00	1,30	9,00
102,00	5,00	10,00	0,00	2,00	18,00	0,93	10,00
96,00	3,00	6,00	0,00	1,00	17,00	1,62	8,00
90,00	4,00	6,00	0,00	1,00	14,00	0,61	10,00
41,00	3,00	3,00	0,00	1,00	5,00	1,38	2,00
90,00	5,00	10,00	0,00	1,00	9,00	0,74	9,00
53,00	3,00	7,00	0,00	1,00	7,00	1,93	2,00
37,00	2,00	3,00	0,00	0,00	1,00	0,78	10,00
18,00	1,00	1,00	0,00	0,00	1,00	0,83	6,00
104,00	4,00	6,00	0,00	1,00	20,00	0,73	10,00
111,00	3,00	8,00	0,00	1,00	19,00	1,41	3,00
81,00	3,00	5,00	0,00	1,00	13,00		10,00
45,00	3,00	6,00	0,00	1,00	8,00	1,18	5,00
76,00	3,00	7,00	0,00	1,00	10,00	0,62	10,00
21,00	4,00	0,00	0,00	0,00	2,00	1,16	1,00
120,00	5,00	10,00	0,00	2,00	24,00	0,89	10,00
78,00	5,00	3,00	0,00	2,00	17,00	1,59	2,00
20,00	1,00	0,00	0,00	0,00	0,00	0,85	9,00
58,00	2,00	7,00	0,00	0,00	2,00		2,00
101,00	4,00	10,00	0,00	1,00	14,00	1,04	7,00
99,00	5,00	6,00	0,00	2,00	19,00	0,78	10,00
91,00	4,00	6,00	0,00	1,00	12,00	0,87	10,00
40,00	2,00	0,00	0,00	0,00	4,00	1,23	6,00
